# Anti-α-amino-3-hydroxy-5-methyl-4-isoxazolepropionic acid receptor GluR2 encephalitis in a myasthenia gravis patient with complete thymectomy: a case report

**DOI:** 10.1186/s12883-019-1358-7

**Published:** 2019-06-13

**Authors:** Qingyang Luo, Xianghong Wu, Wen Huang

**Affiliations:** 1 Department of Neurology, The First Affiliated Hospital of Guangxi Medical University, #6 Shuangyong Road, Nanning, 530021 Guangxi China; 2 Vasculocardiology, First Affiliated Hospital, Guangxi Medical University, Nanning, People’s Republic of China 530021

**Keywords:** Autoimmune encephalitis, α-Amino-3-hydroxy-5-methyl-4-isoxazolepropionic acid receptor, Thymoma, Myasthenia gravis

## Abstract

**Background:**

Autoimmune encephalitis (AE) is a newly recognized autoimmune disorders in which the targets are proteins or receptors involved in synaptic transmission and neuronal excitability. α-amino-3-hydroxy-5-methyl-4-isoxazolepropionic acid receptor (AMPAR) is a subtype of glutamate receptor that mediates most of the fast excitatory neurotransmission in the brain.

**Case presentation:**

A 50-year-old woman presented with subacute onset of memory loss and behavioral changes. High levels of serum (1:1000) and CSF (1:32) antibodies against the AMPAR GluR2 were detected. A wide range of abnormalities in 6–8 Hz low to middle slow waves was found by electroencephalographs, and high-intensity signals on fluid-attenuated inversion recovery in both the medial temporal lobe and hippocampus were identified on brain magnetic resonance images. This patient presented with myasthenia gravis and type B2 thymoma (World Health Organization Thymoma Classification) at age 48. This case was unique in that the patient initiated with the symptom of myasthenia gravis and thymoma two years prior to encephalitis, and a complete thymectomy was performed before AE onset without recurrence of the thymoma when encephalitis occurred.

**Conclusions:**

Thymoma was reported to be associated with paraneoplastic neurological disease. This is the first time a thymectomy has been applied in a myasthenia gravis patient with thymoma two years prior to the onset of anti-AMPAR2 encephalitis. This case highlights the complexity of autoimmune encephalitis associated with thymoma.

## Background

Autoimmune encephalitis (AE), which is characterized by the subacute (days to weeks) development of seizures, recent memory loss, mental confusion and psychiatric symptoms due to antibodies against neuronal cell surface and synaptic proteins, is newly recognized autoimmune disorders involved in synaptic transmission and neuronal excitability [1]. Although thymoma or thymic carcinoma has been reported to be associated with AE, the most common targets are LGI1, Caspr2 and γ-aminobutyric acid (GABA)- A type receptor antibody encephalitis [2–5]. Thymoma-associated encephalitis associated with α-amino-3-hydroxy-5-methyl-4-isoxazolepropionic acid receptor (AMPAR), one of neuronal surface autoantigens, is rare [6]. In this report, we describe a female thymomatous myasthenia gravis (MG) patient with AMPAR encephalitis onset after a complete thymectomy without recurrence of the thymoma.

## Case presentation

A 50-year-old woman presenting with subacute onset of memory loss and behavioral changes lasting for one month was admitted to our hospital on July 9, 2018. On examination, she was confused and apathetic, disoriented to time and space with impaired memory and executive dysfunction, slurred speech with partly comprehensive aphasia, and urinary and fecal incontinence when she was admitted to our hospital. She was poorly collaborative in Mini-Mental State Examination (MMSE). No localized symptoms or signs were observed.

**Fig. 1:**
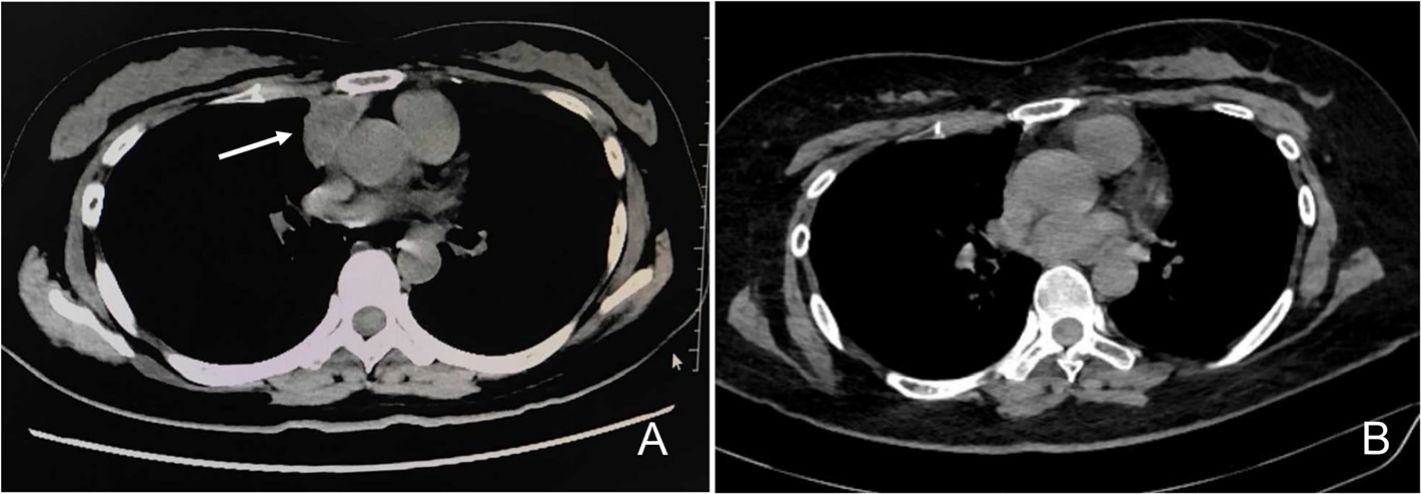
Chest CT showed a 3.1 cm × 1.9 cm thymoma on August 3, 2016 (**a**) and was normal without thymoma recurrence after thymectomy on July 11, 2018 (**b**)

Two years ago, this patient presented at age 48 with right ptosis. She was diagnosed with MG based on positive AChR Ab (titre > 20 nmol/L) and a neostigmine test, as well as a decrement of 15% in low frequency (3 Hz) repetitive nerve stimulation on orbicularis oculi muscles, trapeziuses and deltoid muscles. A computerized tomography (CT) chest showed a thymoma (3.1 cm × 1.9 cm), which was resected on August 22, 2016 (Fig. 1a). Histological examination showed WHO type B2: Kpan(+++), CK19(+++), CD30(+++), CD20(−), CD3(−), CD5(+), TdT(+), and Ki67(+, 90%). Whole-body bone scans by emission computed tomography were normal. Space-occupying lesions were not found in the liver, kidneys or subclavian area investigated with B-ultrasound. For the next two years, the patient’s symptoms were well-controlled with pyridostigmine treatment.

**Fig. 2:**
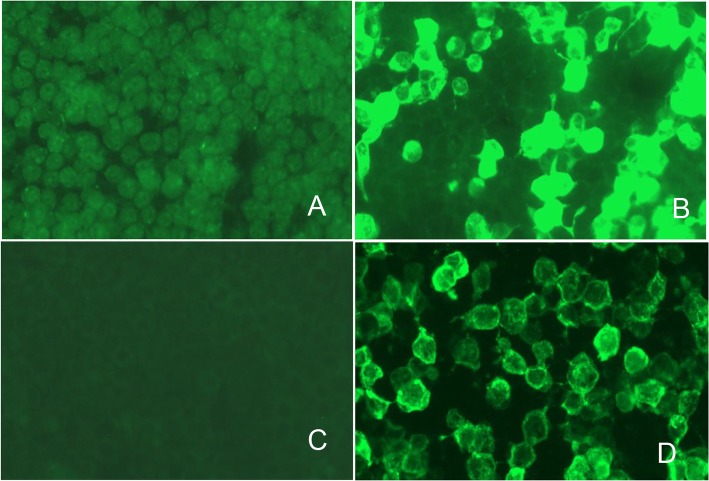
Neuroimmunological investigations showed high levels of serum (1:1000, **b**) and CSF (1:32, **d**) antibodies against the AMPAR GluR2. **a** and **c** were serum and CSF negative control respectively

**Fig. 3:**
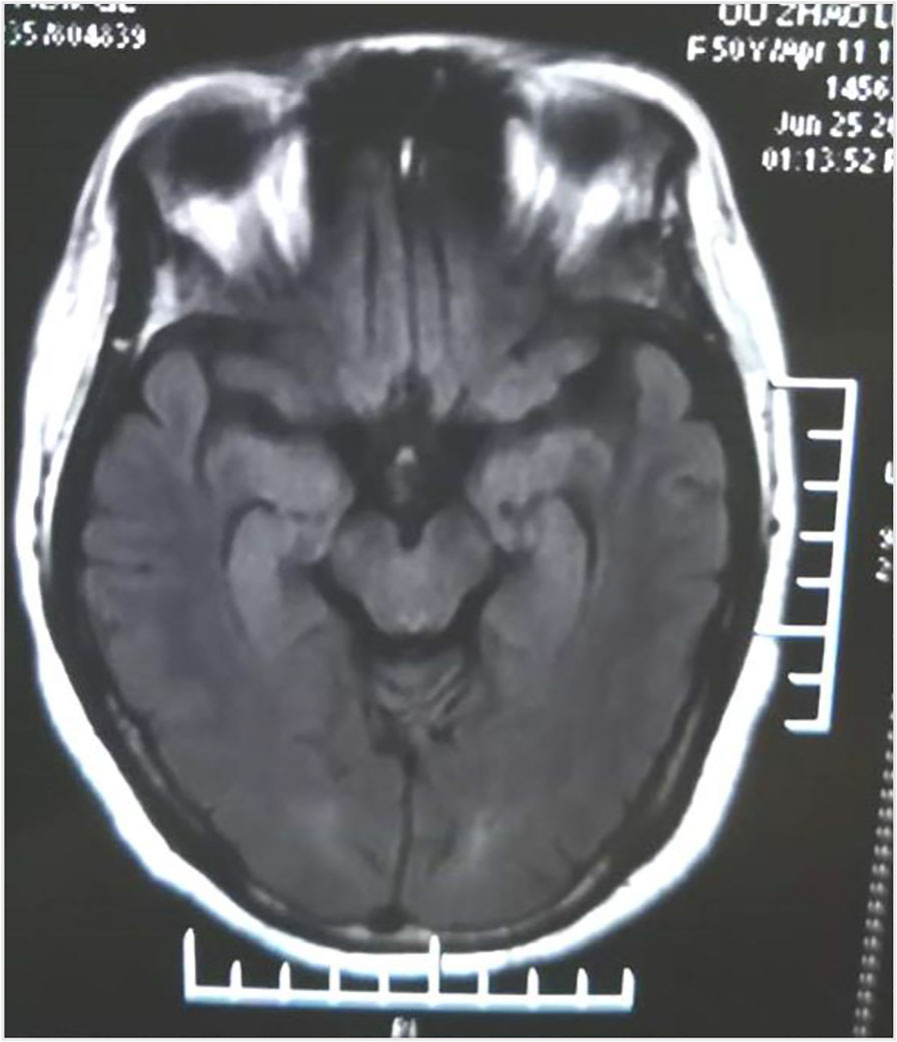
Brain MRI performed on June 25, 2018 identified high-intensity signals on fluid-attenuated inversion recovery in both the medial temporal lobe and hippocampus

Routine serum analyses were within the normal range, including thyroid hormones and associated antibodies, anti-nuclear antibody, and anti-dsDNA. Polymerase chain reaction (PCR) for herpes simplex virus, cytomegalovirus, influenza virus, enterovirus, and measles virus in serum were also negative. Cell count, protein, glucose and chloride were normal in the cerebrospinal fluid (CSF). CSF and serum were negative for oligoclonal bands. Antibodies against cell surface or synaptic proteins were assessed in serum and CSF obtained before immunotherapy using transfected HEK-293 cells by the indirect immunofluorescence method (Kindstar Global, Wu Han, China) showed high levels of serum (1:1000) and CSF (1:32) antibodies against the AMPAR GluR2 (AMPAR2, Fig. 2b and d). A wide range of abnormalities in 6–8 Hz low to middle slow waves was found by electroencephalographs (EEGs). Brain magnetic resonance images (MRI) identified high-intensity signals on fluid-attenuated inversion recovery (Flair) in both the medial temporal lobe and hippocampus on June 25, 2018 (Fig. 3). Antibodies against α-amino-3-hydroxy-5-methyl-4-isoxazolepropionic acid (AMPA) GluR1, N-methyl-D-aspartate receptor, GABA B type receptor, leucine-rich glioma inactivated protein 1 (LGI1) and Caspr2 were negative. The chest CT was normal without thymoma recurrence after thymectomy on July 11, 2018 (Fig. 1b). Ultrasounds of the liver, pancreas, spleen, kidney, bladder, ovary and uterus, as well as serological tumor markers, were also normal and did not reveal a neoplasm. Anti-AMPAR2 encephalitis was thus diagnosed. Intravenous methylprednisolone (1 g/day for 3 days and 500 mg/day for 3 days) followed by oral prednisolone (1 mg/kg/day) was administered with slow tapering. The patient’s symptoms did not have significant remission, and azathioprine (50 mg twice a day) was applied when she was discharged on July 20, 2018. A neuropsychological exam showed memory deficit, calculative and comprehensive dysfunction, lack of motivation and social emotion and urinary incontinence.

The patient had voluntary urinary control, and the initial MRI lesions resolved in 16 days (July 11, 2018). The patient only had mild long-time memory deficits and a good recovery with an MMSE score of 24 at follow-up on September 13, 2018.

## Discussion

AMPAR is a subtype of glutamate receptor that mediates most of the fast excitatory neurotransmission in the brain. The majority of AMPAR are tetramers composed of GluR1, 2, 3 or 4 subunits. A high level of GluR1/2 and GluR2/3 receptors is found in the synaptic CA3-CA1 areas of the hippocampus followed by the subiculum, cerebellum, caudate-putamen, and cerebral cortex [7].

The thymus is a central organ for the development of the immune system, particularly for the selection of T cells with appropriate self-tolerance [8]. It is not surprising that thymoma is considered to be a possible initiator for most paraneoplastic neurological disease, such as AE [8]. In 2009, Lai [7] first reported three patients with anti-AMPAR encephalitis, and thymoma were identified among 109 cases of limbic encephalitis. To date, eleven cases of thymoma-associated anti-AMPAR2 encephalitis have been described in six publications (Table 1) [6, 7, 9–12]. In most cases, thymoma was detected in oncological screening with the first episode of encephalitis, though a malignant thymoma treated with radiotherapy and chemotherapy six years prior to encephalitis was found in one case [9]. Our case was unique in that the patient initiated with the symptom of myasthenia gravis and thymoma two years prior to AE, and a complete thymectomy was performed before AE onset without recurrence of the thymoma when AE occurred. Recently, a patient with anti-AMPAR encephalitis who was reported to be in remission for 34 months showed clinical relapse three months after the detection of recurrent thymoma [11]. Hor et al. reported a case of thymoma-associated myasthenia gravis and LGI1-encephalitis with nephrotic syndrome after thymectomy [13]. Our case raises the questions of whether the autoantigens could be expressed by the thymus after thymectomy, and how these autoantigens from thymic tumors could trigger immune disorders. It is probable that these autoantigens were expressed by the thymus at an early stage before thymectomy and existed in the central nervous system or blood circulatory system and that AE was triggered by these autoantigens due to virus infection or over-fatigue.

**Table 1:** Relation of thymoma and outcome in patients with anti-AMPAR encephalitis

Case	sex/ age	Tumor	Time from symptoms of AE to tumor diagnosis	Other autoimmune or antibodies	Treatment	Outcome	Reference
1	F/44	Thymic carcinoma	Concurrent with first episode of encephalitis	ANA, dsDNA, cardiolipin antibodies	Tumor removal. At presentation and relapse: IVIg, corticosteroids. Chronic treatment with azathioprine.	First episode: returned to baseline. Subsequent relapsing: memory deficit. Residual short-term memory deficit after 3rd relapse.	Lai et al. 2009
2	M/38	Malignant thymoma	Concurrent with relapse of encephalitis	GAD antibodies	Tumor removal, radiation therapy, corticosteroids, plasma exchange, IVIg	First episode: returned to baseline. Mild residual memory deficit after relapse; steroid dependant muscle spasms and rigidity.	Lai et al. 2009
3	F/44	Thymoma	Concurrent with first episode of encephalitis	CV2/CRMP5 antibodies	N/A	Unexpected death due to cardiorespiratory arrest.	Lai et al. 2009
4	F/60	Malignant thymoma	Six years before first episode of encephalitis with residual thymoma without evidence of relapse.	None	Radiotherapy and chemotherapy six years before AE without thymectomy. At presentation: corticosteroids	Complete recovery	Graus et al. 2010
5	F/47	Thymoma (WHO type B1)	Concurrent with first episode of encephalitis	anti-AchR and titin antibodies	Corticosteroid at first episode. Corticosteroid, azathioprine and tumor removal when relapse.	First episode: short term memory deficit, severe anomic aphasia, executive dysfunction.Mild anomic aphasia and bulbar symptoms when relapse.	Li et al. 2015
6	62/M	Malignant thymoma	Concurrent with first episode of encephalitis	None	Tumor removal, corticosteroid, IVIg	Full treatment response	Höftberger et al. 2015
7	23/M	Thymoma	Concurrent with first episode of encephalitis	None	Tumor removal, IVIg, corticosteroid, rituximab	Partial treatment response	Höftberger et al. 2015
8	53/F	Malignant thymoma	Concurrent with first episode of encephalitis	CRMP5	Tumor removal, chemotherapy, radiotherapy, corticosteroid, IVIg	No treatment response	Höftberger et al. 2015
9	71/M	Thymic carcinoid	Concurrent with first episode of encephalitis	NMDAR	Tumor removal, corticosteroid, plasma exchange	Full treatment response	Höftberger et al. 2015
10	21/M	Thymic carcinoid	Concurrent with first episode of encephalitis	None	Tumor removal, chemotherapy, radiotherapy, corticosteroid, IVIg	Mild deficits in working memory persisted at 18 months after disease onset.	Joubert et al. 2015
11	F/34	Thymoma (WHO type B3)	Concurrent with first episode of encephalitis; Recurrence with clinical relapse	None	Corticosteroid and tumor removal at first episode. Corticosteroid and resection of the recurrent tumor after relapse.	First episode: depressive symptoms. Memory deficits after relapse.	Omi et al. 2018
12	F/50	Thymoma (WHO type B2)	2 years before first episode of encephalitis	anti-AchR antibodies	Tumor removal 2 years before AE. Corticosteroid and azathioprine.	First episode: returned to baseline.	Huang et al.

Abbreviations: *N/A* not applicable, *IVIg* intravenous immunoglobulin

Fourteen cases in 22 patients with anti-AMPAR encephalitis had tumors, such as lung cancer, thymoma, breast cancer, or ovarian teratoma, indicating that paraneoplastic autoimmunity plays a key role in the pathogenic mechanisms of anti-AMPAR encephalitis [10, 14]. Our case is more likely paraneoplastic autoimmune encephalitis even if encephalitis emerged 2 years after thymoma diagnosis.

Anti-AMPAR encephalitis has been reported to be associated with other autoimmune antibodies, such as antibodies against ANA, dsDNA, cardiolipin, glutamic acid decarboxylase (GAD), CV2/CRMP5 and AChR [5, 6]. AChR Ab is also positive in our patient; however, the immune relationships between these antibodies and AMPAR Ab has not been thoroughly elucidated to date.

Based on the clinical outcome of our patient and review of the published literature, tumor removal with or without chemotherapy and radiotherapy may be applied to anti-AMPAR encephalitis with thymic tumors, and immunotherapy including corticosteroids, IVIg and plasma exchange during presentation or relapse and chronic treatment with rituximab or azathioprine may prevent relapse and improve outcomes. In patients with anti-AMPAR encephalitis, oncological screening is mandatory with a particular focus on the thymus, lung and breast [12].

There are several limitations to our study. A more intense oncological screening and long-term follow-up is needed to determine whether the patient has other tumors or metastatic tumors in addition to thymoma.

In conclusion, we presented a case in which a thymectomy was applied in a myasthenia gravis patient with thymoma two years prior to the onset of anti-AMPAR2 encephalitis. This case highlights the complexity of autoimmune encephalitis associated with thymoma.

## Data Availability

Data generated during this study are included in this published article.

## Abbreviations

AE: Autoimmune encephalitis
AMPAR: α-amino-3-hydroxy-5-methyl-4-isoxazolepropionic acid receptor
AMPAR2: AMPAR GluR2
CSF: Cerebrospinal fluid
Flair: Fluid-attenuated inversion recovery
GABA: γ-aminobutyric acid
MG: Myasthenia gravis
MMSE: Mini-Mental State Examination
